# Washington State landowner data on cropping decision factors and perceptions of bioenergy crops

**DOI:** 10.1016/j.dib.2018.02.077

**Published:** 2018-03-08

**Authors:** Catherine H. Gowan, Shiba P. Kar, Patricia A. Townsend

**Affiliations:** Washington State University, University of Wisconsin- Stevens Point, United States

## Abstract

This article contains survey data on Washington State landowner perceptions of bioenergy crops. The survey data includes responses on interest in growing bioenergy crops, likelihood of growing bioenergy crops, and willingness to grow hybrid poplar specifically for bioenergy. Additional responses concern challenges to growing hybrid poplar for bioenergy and preferences for growing hybrid poplar versus other bioenergy crops. The data contains on farm information, landowner demographics, current crops and cropping decisions, and perceptions of perennial crops as well. Responses were collected from 156 randomly sampled landowners with land appropriate for growing hybrid poplar trees without irrigation.

**Specifications Table**

**Table:** 

Subject area	Social science
More specific subject area	Stakeholder research
Type of data	Excel file
How data was acquired	Survey
Data format	Raw
Experimental factors	Not applicable
Experimental features	Not applicable
Data source location	Washington State
Data accessibility	Not in a public repository
Related research article	Gowan, C. H., Kar, S.P., and Townsend, P.A. Landowners’ perceptions of and interest in bioenergy crops: Exploring challenges and opportunities for growing poplar for bioenergy. 2018. *Biomass and Bioenergy.* 110. 57–62. DOI: https://doi.org/10.1016/j.biombioe.2018.01.015

**Value of the Data**

• Serves as a benchmark of landowner attitudes and perceptions of bioenergy crops.
• Provides information about the perceived importance of current and bioenergy cropping decision factors.
• Only known survey of Washington State landowners about growing hybrid poplar for bioenergy.
• Provides demographic data of randomly selected landowners in areas of Washington State with land suitable for growing poplar.
• Provides potential comparisons to research conducted in other states on landowner interest and likelihood of growing bioenergy crops.

## 1 Data

The data is from a 38-question survey administered to randomly sampled landowners in Washington State. It includes responses about their interest in growing bioenergy crops, likelihood of growing bioenergy crops, and willingness to grow hybrid poplar for bioenergy. The data includes landowner demographics, farm information, current crops, cropping decision factors, and comparisons between various bioenergy crops. The data is in the form of Likert-like scales, binary, categorical, ordinal, and numerical responses. The original question text is included for each variable.

## 2 Experimental design, materials, and methods

The survey was designed collaboratively with Advanced Hardwood Biofuels Northwest Extension staff, Washington State University's Social and Economic Sciences Research Center, and faculty at the University of Washington and Washington State University who had been conducting qualitative research with landowners.

Contact information for landowners was collected through a Washington statewide geographic information system landowner database. Researchers at the University of Washington created a land parcel database for Washington State and then conducted a suitability study to determine the areas appropriate for poplar growth [1]. Land parcels that were highly or moderately suitable for growing poplar without irrigation were included in the sample. The sample was selected from two categories of lands: (a) resource production and extraction, (b) undeveloped land. All land parcels were larger than 20 acres. Federal, state, agency, trust, or organization-owned land and developed land were not included.

This process yielded 40,000 parcels, of which 1050 parcels were randomly selected and landowner contact information obtained. Stratified random sampling was used to ensure that landowners with small (20–40 ac.), medium (41–160 ac.), and large (>160 ac.) parcels of land would be represented. After removing incorrect contacts, introductory letters were sent to 900 landowners in two phases.

Introductory letters were personalized with the respondent's name and address and were printed on Washington State University College of Agriculture, Human and Natural Resources Letterhead. Letters included instructions for completing the survey online along with the survey URL (or web survey address) and the respondents’ unique access code for accessing the web survey. If the respondent did not answer the survey online, they were mailed a paper questionnaire. All paper questionnaires mailed included a respondent ID number (the same as the internet survey access code) to track whether a questionnaire was completed and returned or completed on the web. A self-addressed postage paid envelope was included with each paper questionnaire. The mailing sequence varied slightly between Phase One and Phase Two (Table 1).

**Table 1:** Survey contact schedule.

**Phase One Contact Type**	Date
Introductory letter with web link and unique access code	April 8, 2014
Postcard reminder with web link and unique access code	April 16, 2014
Reminder letter with web link, access code, paper survey, and postage paid return envelope	April 25, 2014
Final postcard reminder postcard with web link	May 15, 2014

**Phase Two Contact Type**	Date
Introductory letter with web link and unique access code, return postcard	November 7, 2014
Cover letter with web link, access code, paper questionnaire, postage paid return envelope	November 12, 2014
Second letter with web link, access code, replacement questionnaire, postage paid return envelope	December 1, 2014

In Phase Two, a postage paid return postcard was included in the mailing of the introductory letter. The postcard gave respondents the opportunity to provide an alternative name and contact information for the “person who makes decisions about crops on the farmland” and to mail back that information to Social and Economy Sciences Research Center. When a new name and address was provided, that sample record was updated and survey mailings were sent to the new name and address.

The Phase One and Phase Two data collection periods were held open for approximately 10 weeks each to allow for return of mail questionnaires in response to the four mail contacts. Data collection receipt was closed off on June 15, 2014 for Phase One and January 25, 2015 for Phase Two. One hundred and fifty-six questionnaires were completed and returned.
